# Using a Sweating Residuum/socket Interface Simulator for the Evaluation of Sweat Management Liners in Lower Limb Prosthetics

**DOI:** 10.33137/cpoj.v4i1.35213

**Published:** 2021-03-19

**Authors:** M McGrath, K.C. Davies, A Gallego, P Laszczak, J Tang, S Zahedi, D Moser

**Affiliations:** 1 Blatchford Group, Unit D Antura, Bond Close, Basingstoke, UK.; 2 School of Engineering, Faculty of Engineering and Physical Sciences, University of Southampton, UK.

**Keywords:** Sweating, Residual limb, Socket interface, Simulator, Lower limb prosthetics, Amputation

## Abstract

**BACKGROUND:**

Lab-based simulators can help to reduce variability in prosthetics research. However, they have not yet been used to investigate the effects of sweating at the residuum-liner interface. This work sought to create and validate a simulator to replicate the mechanics of residual limb perspiration. The developed apparatus was used to assess the effects of perspiration and different liner designs.

**METHODOLOGY:**

By scanning a cast, an artificial residuum was manufactured using a 3D-printed, transtibial bone model encased in silicone, moulded with pores. The pores allowed water to emit from the residuum surface, simulating sweating. Dry and sweating cyclic tests were performed by applying compressive and tensile loading, while measuring the displacement of the residuum relative to the socket. Tests were conducted using standard and perforated liners.

**FINDINGS:**

Although maximum displacement varied between test setups, its variance was low (coefficient of variation <1%) and consistent between dry tests. For unperforated liners, sweating increased the standard deviation of maximum displacement approximately threefold (0.04mm v 0.12mm, p<0.001). However, with the perforated liner, sweating had little effect on standard deviation compared to dry tests (0.04mm v 0.04mm, p=0.497).

**CONCLUSIONS:**

The test apparatus was effective at simulating the effect of perspiration at the residual limb. Moisture at the skin-liner interface can lead to inconsistent mechanics. Perforated liners help to remove sweat from the skin-liner interface, thereby mitigating these effects.

## INTRODUCTION

Excessive sweating commonly affects lower limb amputees^1^ and impacts their daily life.^2^ Increased energy expenditure during everyday activities compared to able-bodied people^3^ and reduced skin surface area^4^ for cooling both contribute to this issue. Prosthetic liners worn on the residuum can also amplify sweating at the residuum-liner interface, as they have poor thermal conductivity^5^ and little permeability.^6^

The socket and residual limb are often considered a single entity with a rigid connection. However, in practice, there is relative movement at this interface,^7,8^ which sweating can worsen, affecting prosthetic suspension.^9^

Technologies have been developed to regulate residuum temperature or manage perspiration.^10–13^ One such technology uses perforations in the liner to allow sweat to transfer away from the skin. Previous evaluations of this technology have reported higher scores in patient-reported outcome measures,^13^ fewer skin health problems^12,13^ and a noticeable reduction in the perspiration on the limb.^12,13^

Due to the inherent heterogeneity and variability of amputees,^14^ some researchers have emulated the residual limb using simulators and test machines, in lieu of human participants.^15,16^ These can recreate realistic interface mechanics in prosthetic sockets,^17–20^ in a highly reproducible and repeatable manner. These methods have not yet been used to examine effects of perspiration at the residuum-liner interface.

### Objectives

This research sought to achieve the following objectives; design, construct and evaluate a test apparatus to recreate the impact of sweat at the residuum-liner interface, identify how displacement during loading is affected by the presence of moisture, and evaluate the efficacy of a liner designed for perspiration management.

## METHODOLOGY

### Manufacture

This research followed a similar artificial residuum manufacturing method to McGrath et al.^17^ A transtibial residuum cast was scanned and a pin-lock check socket was created. A transtibial bone model of an extended knee was also scanned so that the bones and residuum could be scaled in size to match one another. The scaled bone model and two halves of a negative residuum mould were created using additive manufacture. The soft tissue was simulated by moulding silicone (Smooth-On, Inc., PA, USA; density = 1.08 g/cm3) around the bone model.

**Figure 1:**
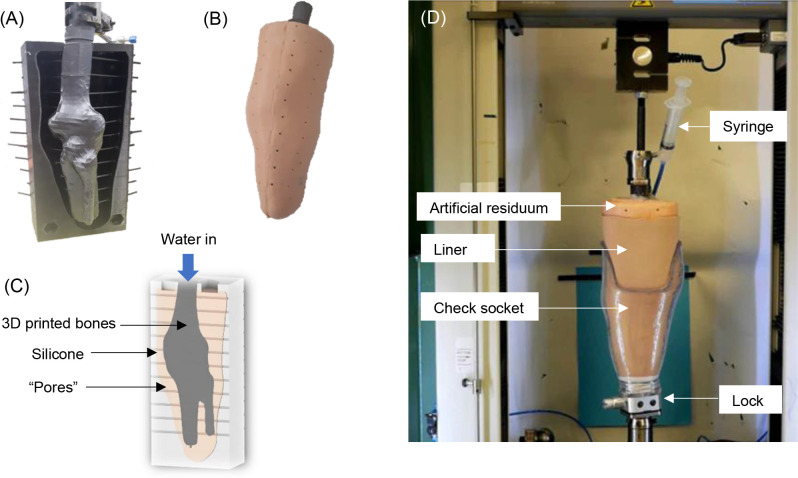
(**A**) The 3D printed bones held in place in the negative residuum mould with 3mm straws to create “pores” (**B**) the moulded silicone artificial residuum with pores visible along its surface (**C**) an annotated cross-section diagram of the artificial residuum (**D**) the artificial residuum set up on the test machine.

During moulding, 3mm diameter plastic straws were used to create pores in the silicone, evenly spaced along the length and around the circumference, with one at the distal end (Figure 1A). The 3mm diameter was the minimum that could be consistently 3D printed.

Water was applied, via a syringe and rubber tubing, into the proximal opening between silicone and bone. Once in the central canal, applying compression forced the water through the pores to the outer surface. The proximal hole was sealed with the rubber tubing in place, using a silicone adhesive. The residuum and its cross-section are shown in Figures 1B and 1C, respectively. A female pyramid tube adaptor was fixed to the proximal end of the bone model allowing rigid attachment to a universal test machine (LR10K Plus^i^, Lloyd Instruments, UK – Figure 1D).

### Protocol

The residuum was fitted with a pin-lock liner (Comfort liner^ii^, Blatchford Ltd, UK) and attached to the check socket. Since this simulator sought to mimic both stance and swing phase, for simplicity, it was vertically-oriented on the test machine (Figure 1D).

Tests were load controlled. A single cycle increased to a maximum 800N compression, reversed to a maximum 100N tension, then returned to 0N. 800N is a typical maximum force applied by an adult of approximately 70-80kg during walking.^21^ 100N is a liberal estimate of the combined peak gravitational and centripetal forces on the limb during swing phase.

The outcome measurement was displacement, recorded by the actuator of the test machine. The measurement was zeroed before any compression occurred, so the output reflected the change in displacement of the proximal attachment of the residual limb, from its initial position, due to the loading pattern.

For sweating tests, 20ml of water was added, based on an approximation calculated from reported amputee sweating rates^22^ and the residuum surface area. Following each sweating test, the liner was removed to note the quantity of water remaining inside and the residuum was heated for eight hours in an oven at 40°C to ensure the evaporation of any residual water. The order of testing for dry and sweating tests was randomised.

### Repeatability

Tests were 50 seconds long, performing 50 cycles at a frequency of 1Hz to simulate a 120 steps per minute walking cadence. Of typical walking bouts, 75% consist of fewer than 40 steps and 60% last under 30 seconds,^23^ so each simulator test would represent the majority of these bouts.

### Reproducibility

It was possible that deconstruction/reassembly of the setup would create differences in the exact fit of the liner on the residuum or the residuum in the socket. This replicates the real-world conditions of doffing and donning a prosthesis day-to-day. Three dry tests of 50 cycles each were performed and the simulator was deconstructed and reconstructed between tests, to quantify this effect.

### Liners

The protocol was used to evaluate perforated liners (Silcare Breathe Locking^iii^, Blatchford Ltd, UK). These liners have perforations along the length (columns of 150), circumference (eight columns) and at the distal end (60) to allow sweat removal. The perforated and unperforated liners were made with the same silicone, the same fabric (polyamide and lycra) and the same thickness profile (7mm distally, 2.9mm proximally), so the only difference was the perforations. The order of liner testing was random.

### Data processing

The first five recorded cycles of each test were excluded from data analysis to account for any human error during setup e.g. the pin ratcheting further into the lock. The remaining 45 cycles were checked to ensure at least 780N compression. Displacement values were compared between tests by magnitude (mean values) and variability (standard deviation (SD), coefficient of variation (CV)). Shapiro-Wilk tests evaluated data normality. The Brown-Forsythe test for homogeneity of variance determined whether datasets had equal variances. This test was chosen for its robustness with non-normal distributions. For normal data, t-tests compared mean displacements. For non-parametric data, Wilcoxon tests were used if group variances were homogenous, otherwise Kruskal-Wallis tests were employed. Statistical significance was defined as p≤0.05.

## RESULTS

### Reproducibility

**Figure 2:**
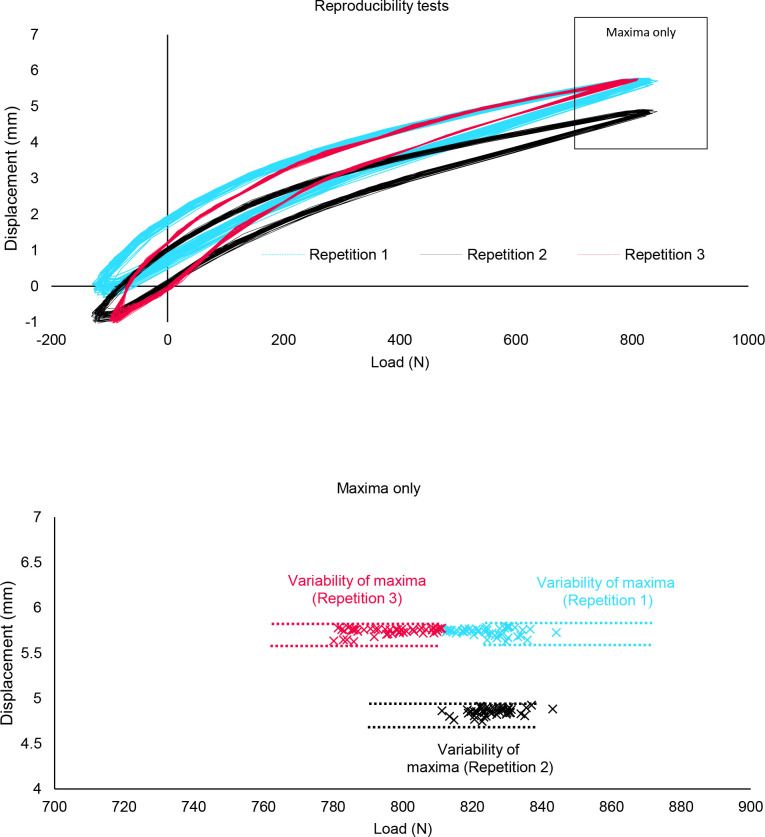
(Top) The displacement v load curves for the three reproducibility tests. Positive load and displacement indicate compression, negative load and displacement indicate tension. N.B. Not all curves pass through the origin due to the exclusion of the first five cycles. (Bottom) The maximum displacements of each of the cycles, for each reproducibility test. The variability of these maxima, within each test, is annotated.

Hysteresis curves for the three reproducibility tests are shown in Figure 2. Measurements showed that absolute displacement was sensitive to the simulator setup. Maximum values for each repetition (mean±SD; 5.73±0.04mm, 4.85±0.04mm, 5.78±0.04mm, respectively) showed a statistically significant difference (p<0.001). Minimum values for each test (-0.07±0.13mm, -0.86±0.10 mm, -0.95±0.06mm, respectively) also showed a statistically significant difference (p<0.001).

### Repeatability

The CVs for maximum displacements were 0.7%, 0.8% and 0.6% for Repetitions 1, 2 and 3, respectively. The Brown-Forsythe test indicated no significant difference in the variances of these tests (p=0.42). Variability of maximum displacement was chosen to compare between further tests.

### Sweating

**Figure 3:**
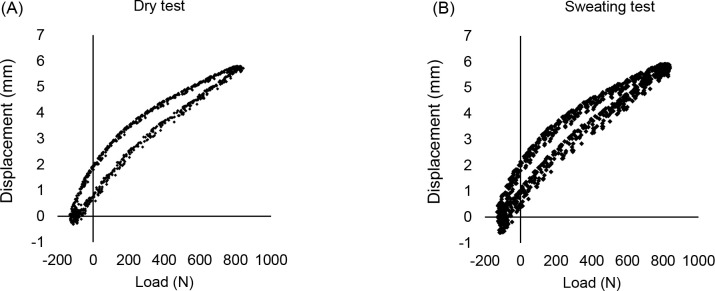
(A) The hysteresis curve of a dry test with the standard liner (B) the hysteresis curve of a ‘sweating’ test with the standard liner.

Figure 3A and 3B show the hysteresis curves of a dry test and a sweating test for a standard liner. Maximum displacement increased with each cycle of the sweating test. The SD of maximum displacement of the sweating test (0.12mm) was significantly higher than for the dry test (0.04mm, p<0.001).

After the sweating test approximately 50% of the water applied was poured out from the bottom of the liner. This did not include any water that may have been remaining on the artificial residuum or in its pores.

### Liners

**Figure 4:**
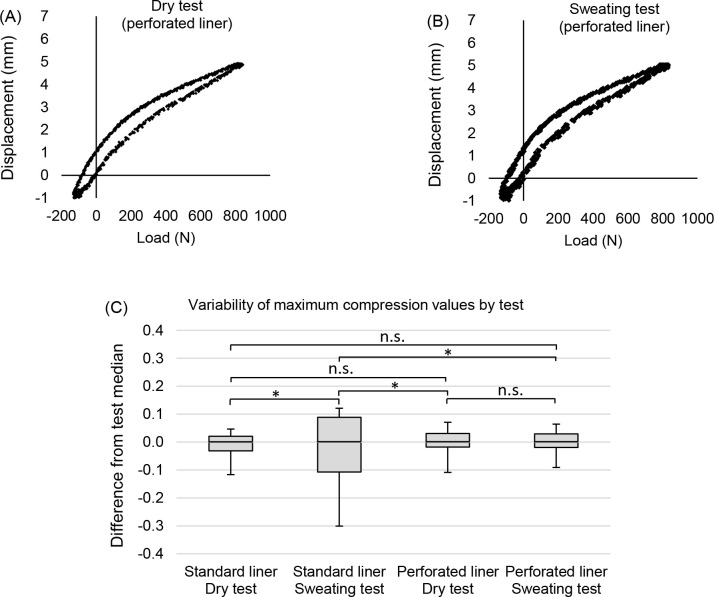
(A) The hysteresis curve of a dry test with the perforated liner (B) the hysteresis curve of a sweating test with the perforated liner (C) A box-and-whisker plot of the maximum displacement values from the dry and sweating tests with the standard and perforated liners (normalised by median). The box indicates the interquartile range and the whiskers indicate the maximum and minimum values. The lines at the top of the plot show where comparisons of variability were made. Asterisks (*) indicate a significant difference (p<0.05) in variance between tests.

Figure 4A and 4B show the differences in hysteresis curves between a dry test and a sweating test for a perforated liner. The sweating test retained a high degree of repeatability (CV=0.7%), comparable to the dry tests. The variability of the sweating test (SD=0.04mm) and the dry test (SD=0.04mm) were not significantly different (p=0.497).

Figure 4C, shows the interquartile ranges for all tests, normalised by medians. The variability of the sweating test with the perforated liner (SD = 0.04mm) was significantly less than with the standard liner (SD=0.12mm, p<0.001).

After the test, the volume of water poured out from the liner was approximately 5% of the volume originally applied. This did not include any water that may have been (A) remaining on the surface of the residuum, (B) in its pores, (C) absorbed by the outer liner fabric, or (D) expelled from the socket at the distal end, via the lock, which could not be accurately quantified.

## DISCUSSION

A simulator was successfully constructed to mimic the interface dynamics of a sweating residual limb. Tests demonstrated that moisture at the residuum-liner interface leads to greater variability in the displacement of the residual limb, relative to the socket, when loaded to replicate walking. When a perforated prosthetic liner was used to allow moisture to transport away from the interface, the variability of displacement was equivalent to that of dry tests, under the same walking load pattern.

For simplicity and the constraints of the test equipment, the simulator was mounted vertically on the test machine. While axial displacement is the largest in magnitude^7,24^ and widest-researched,^20,25–28^ the other five degrees-of-freedom (anterior-posterior and medial-lateral translation, as well as rotation about each of the three axes), are also likely to be affected.^7,28^ Regardless, the results demonstrated a sufficient mechanism to identify the influence of perspiration at the liner interface.

In terms of repeatability, the simulator had CVs<1% between strides. Even between reproducibility tests, which were statistically different, differences in maximum displacement were approximately 1mm, and therefore unlikely to be perceptible by a wearer. Consistent suspension is important with suspension method^25–30^ and socket fit/design^31–34^ affecting prosthetic performance.

The effect of sweating was illustrated in Figure 3. Variability (SD) increased approximately threefold (p<0.001); maximum displacement increasing with each progressive cycle. This movement contributes to skin damage^35^ and explains why sweat affects gait quality.^9^ A review of gait stability in non-amputees observed that inconsistent gait parameters were the strongest distinguishing factor between fallers and non-fallers,^36^ with similar observations reported for transtibial amputees.^37^

The effect of using a perforated liner was investigated (Figure 4). While variability increased 194% with the standard liner during sweating tests, there was no significant difference in variability of the dry and sweating tests with the perforated liner (p=0.497). Notably, even when sweating, the perforated liner retained the consistent mechanics of a dry interface.

### Limitations

The scope of the simulator was to develop a method to distribute liquid across the residuum-liner interface. This simplified some characteristics of the residual limb, such as the size and distribution of sweat pores and the heterogeneity of the soft tissue. Nor was it designed to account for the rate of sweat production; the liquid was present from the first loading cycle. Similarly, other conditions associated with sweating (e.g. increased temperature) were not considered in the design.

There were limitations of this simplified design. By adding the liquid at the top there was no way to ensure that all of the water had been pushed to the surface. Furthermore, during the ‘sweating’ test with the perforated liner, water was observed being emitted from the perforations but was not evenly across the liner, perhaps implying that the perspiration was not distributed evenly across the residuum surface. The variable pore length due to residuum geometry and the effect of gravity likely had an impact.

Another potential limitation was the coefficient of friction (CF) between the materials used. The CF between human skin and silicone is between 0.35 and 1.16, with a mean value of 0.6138. The CF of the silicone used to create the artificial residuum is not reported by the manufacturer. However, by keeping it constant between tests, relative comparisons can be made.

An alternative might have been to perform in-vitro experiments with animal specimens. The advantages would have been closer approximations of the mechanical and frictional properties of human tissue. The drawbacks would have been losing the geometry of a residual limb in a socket and less control over the quantity of liquid at the interface.

Finally, it should be noted that these same results may not be generalizable to other liner designs. Differences in the size, profile and distribution of perforations, as well as liner profile and the external fabric may all have an effect on the efficiency of sweat removal.

## CONCLUSION

In conclusion, the test apparatus was effective at simulating perspiration at the residual limb with reproducible results. Perforated liners remove perspiration from the residuum-liner interface, helping to maintain consistent mechanical behaviour. Minimising unwanted movement reduces the risk of soft tissue injury.

## DECLARATION OF CONFLICTING INTERESTS

Some of the authors are full time employees of the manufacturer of the prosthetic liners evaluated in this study

## AUTHOR CONTRIBUTION

**Michael McGrath:** Conceptualisation, manufacturer, data collection, data analysis, writing original, review and editing

**KC Davies:** Writing original, review and editing

**Ana Gallego:** Conceptualisation, manufacturer, data collection, review and editing

**Piotr Laszczak:** Conceptualisation, review and editing

**Jinghua Tang:** Review and editing

**Saeed Zahedi:** Review and editing

**David Moser:** Review and editing

## SOURCES OF SUPPORT

Some of the authors are employees of Blatchford Products Ltd.

## ETHICAL APPROVAL

Ethical approval was not needed for this study.

## MANUFACTURERS’ DOCUMENTATION

i) https://www.ametektest.com/-/media/ametektest/download_links/data_dual_column_test_stands_lr10kplus_data_sheet_english.pdf

ii) https://www.blatchfordus.com/products/comfort-liner/

iii) https://www.blatchfordus.com/products/silcare-breathe-locking-liner/
